# Practical Points

**Published:** 1896-08-08

**Authors:** 


### PRACTICAL POINTS.

### Titles of Staff in Hospital Reports.

*We have received a letter in regard to a recent appointment of an additional honorary dental surgeon to the Montgomeryshire Infirmary. From this it would appear that the gentleman in question was appointed by resolution at an annual meeting; that during the year following his appointment he did not attend at the infirmary; that his name appears in the annual report, followed by the letters R.D.S., D.S.G.B., letters which are not to be found in the list of qualifications published by authority of the General Medical Council at the beginning of the Dentists' Register, and the assumption of which it is stated is likely to convey the impression that they represent qualifications obtained as the result of study and examination when such is not the case. The writer asks whether it is consistent with his duty as a member of an honourable profession to remain on the staff with this gentleman as a colleague.*

To this we would answer that if the account given is correct the infirmary seems to be badly managed. No institution should appoint its honorary staff by resolution at an annual meeting, nor should it allow its officers to neglect their work, nor should it hand over the preparation of its annual report to persons so illiterate as to allow the addition of misleading and unauthorised letters to the names of members of its honorary staff. To all these things the attention of the board of management may properly be called, but so long as a man is legally elected and is on the Register the follies of the Board give no excuse for refusing to co-operate with him as a colleague.

### The House Surgeon's Fees!

*A house surgeon asks a question which raises points of some interest. In his professional capacity as house surgeon he saw a child who had been caned at school. He then received a letter from the clerk to the School Board, asking his opinion regarding the extent of the injury, and after that a second letter asking still more particularly whether the injury was more likely to have been caused by a brick or a stick. To both these letters the house surgeon (who is bound down not to do private practice) answered, giving his opinion on the points raised. He then sent in a bill for a fee of £1 1s. Can he legally claim this fee?*

We cannot enter into the law of the matter, but to us it seems clear that, putting on one side all questions of "private practice," it would be improper, however legal, for a house surgeon to claim a reward for services rendered in regard to an infirmary patient, other than for those services which he is forced to render by law (not necessarily for the patient's benefit) in the way of giving evidence in a court of justice, and for which the law prescribes a certain fee. We would also point out that in our opinion the house surgeon was by no means bound to answer the questions put to him; and bearing in mind the comments which have recently been made in our columns and in other journals in regard to the obligation of professional secrecy, we can have no doubt that he will see that, in giving these details in regard to his patient, he went beyond what he was called upon to do. The public sadly require educating in this matter, and although we have no doubt that it never occurred to the clerk to the School Board that he was making an outrageous request when he applied to the house surgeon for information about his patient, and although the house surgeon clearly fell into the trap quite unwittingly, yet it is clear that a professional rule was violated in giving the information asked for. House surgeons are often placed in extremely awkward positions, and it is as well to be careful not to admit even the thinnest end of the wedge in matters of this kind.

